# Fast and easy visualization of blood flow patterns in 4D Qflow MRI

**DOI:** 10.1186/1532-429X-14-S1-W46

**Published:** 2012-02-01

**Authors:** Vikas Sinha, Gilion Hautvast, Jeroen Sonnemans, Hubrecht de Bliek, Andrei Jalba, Marcel Breeuwer

**Affiliations:** 1 MR Advanced Solutions, Philips Healthcare, Best, Netherlands; 2 Biomedical Image Analysis, Eindhoven University of Technology, Eindhoven, Netherlands; 3 Mathematics and Computer Science, Eindhoven University of Technology, Eindhoven, Netherlands; 4 Clinical Informatics Solutions, Philips Healthcare, Best, Netherlands; 5 MR Clinical Science, Philips Healthcare, Best, Netherlands

## Summary

To enable efficient fast and easy visualization of blood flow patterns in 4D Qflow MRI we have automated vessel segmentation and flow pattern visualization. The new methods enable flow pattern visualization within 10 seconds. As such, our method allows for routine clinical use for flow pattern visualization.

## Background

Four-dimensional Quantitative Flow (4D Qflow) MRI acquisitions result in large data sets, consisting of a set of volume acquisitions taken at equidistant time-intervals over the cardiac cycle. These data sets are preferably analyzed by advanced visualization of flow patterns in conjunction with renderings of the vessel anatomy, which is often difficult and time-consuming to perform. Therefore, our purpose is to automate segmentation and visualization of blood-flow patterns associated with anatomy in a 4D Qflow MRI acquisition.

## Methods

For interactive vessel segmentation, a Temporal Maximum Intensity Projection (TMIP) volume is computed by aggregating the maximum value per voxel over all acquired phases. The TMIP reveals vessel structures based on the magnitude of the phase contrast data, enabling the user to identify a vessel of interest by means of a single mouse click. At the indicated location, the automated segmentation produces a cross section of the lumen and a longitudinal section following the centerline of the vessel path. A ‘ring’ around the lumen is displayed on the TMIP at the selected location.

The velocity data from the cross-section plane is used to compute advanced visualizations such as particle traces and streamlines. Particle traces show the movement of a particle (of fluid) over time, with a trail formed by previous particle positions. The path is computed by integrating the velocity starting from the lumen cross-section. This is done by finding the series of next positions (based on velocity at current position) after a fixed time-step in the image sequence. The velocity at a particular time and position is obtained using Mitchell-Netravali cubic spline interpolation (for all four dimensions). The fourth-order Runge-Kutta numerical scheme is used for the integration calculations. Similar methods are used for streamline construction (instantaneous or fixed velocity field) and pathlines (time-varying velocity field). The direct volume rendering of the gradient in the TMIP volume is used to display an anatomical vessel envelope, overlaid on the advanced renderings, thus showing the underlying flow pattern.

## Results

**Figure 1:**
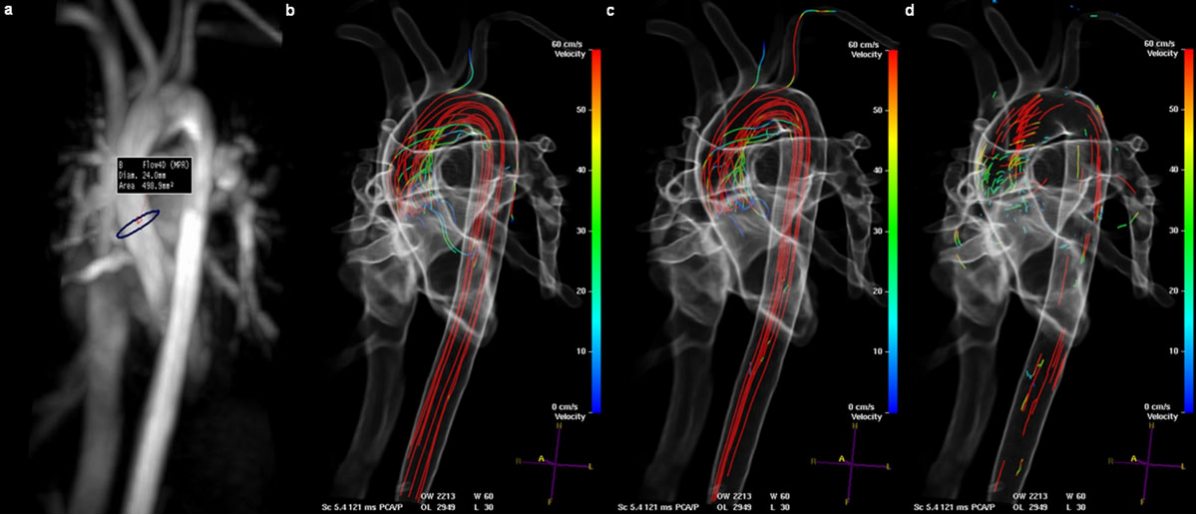
Example results of the a) TMIP, b) pathline visualization, c) streamline visualizaiton and d) particle traces, positioned using a single user interaction.

Figure 1 shows an example of the ’ring’ in the TMIP, pathlines, streamlines and particle visualization, together with direct gradient-based volume rendering. These results are obtained within 10 seconds after loading the 4D Qflow data (on a dual-core 2.33 GHz PC with 3.5 GB RAM).

## Conclusions

Extensive automation enables easy and time-efficient assessment of large vessel anatomy and blood flow patterns by means of advanced visualization of 4D Qflow MRI, within the time constraints of clinical routine.

## Funding

None.

